# Model of Organizational Commitment Applied to Health Management Systems

**DOI:** 10.3390/ijerph18094496

**Published:** 2021-04-23

**Authors:** Mercedes Rodríguez-Fernández, Juan Herrera, Carlos de las Heras-Rosas

**Affiliations:** Department of Economics and Business Administration, Universidad de Málaga, 29071 Málaga, Spain

**Keywords:** organizational commitment, healthcare, nurses, patients, job satisfaction, SciMAT

## Abstract

In this paper, we try to build on the problems surrounding the management of human resources in health care organizations worldwide. After the analysis of the reviewed literature, we detected that the scientific community considers several recurring themes that need attention: stress, burnout, and turnover intention. Based on this, we developed a model of organizational commitment that aims to achieve performance and health quality, its main result the establishment of the appropriate management policies in order to avoid the abandonment of the organization through the search for commitment and job satisfaction. Amongst our main conclusions, we highlight the need to implement a human resources model for hospital administrators based on the relationships with “patients” not “clients” through the maintenance of a positive and strong atmosphere of staff participation. It is important to develop innovative practices related to clear job design that eliminate reasons for ambiguity and stress in executing the tasks of the healthcare system. Finally, we urge training programs in transformational leadership to promote the well-being and organizational commitment of employees.

## 1. Introduction

Healthcare organizations are facing a major challenge with regard to human resource management. The World Health Organization’s (WHO) World Health Report 2006 [1] already warned of the worrying staff shortages faced by hospitals and health centers worldwide and the difficulties in managing them. More recently, the 2030 Agenda for sustainable development goals (SDGs) report [2] indicates that nursing staff is understaffed and unevenly distributed. Healthcare institutions face multiple challenges, characterized by several factors that have been widely reported in the scientific literature. These include increasing societal expectations on these organizations, together with continuous technological and clinical advancement [3]. Another indicator that has been extensively discussed is the adequate management of human resources. Healthcare institutions, like other organizations, need to have good professionals to achieve their objectives, although the treatment of patients and the expected quality of medical care mean that the appropriate management of human resources takes on greater importance in these organizations [4].

As we will see in the development of this manuscript, the scientific community has addressed multiple and varied issues related to employees in healthcare institutions, especially in relation to job satisfaction. Stress [5], burnout [6], burnout syndrome [7,8,9], intention to quit [10,11], and other very visible peculiarities in this sector have concerned both the scientific community and health center administrators [12]. Healthcare institutions are facing a complex situation in relation to human resource management [4]. The analysis of the organizational commitment developed by employees, and its connection with the fulfilment of the psychological contract and their job satisfaction, is positioned as a key tool for any organization, and essential in healthcare institutions due to their special characteristics [8,13].

The extensive scientific production related to human resource management and organizational commitment in healthcare institutions has prompted us to carry out a systematic analysis of published research. The main objective of this research is to inspect exhaustively the publications in this field, with a double purpose: on the one hand, to highlight the main topics on organizational commitment in healthcare institutions; and on the other hand, once the main topics have been grouped, to elaborate a model that gathers the relationships between the different key factors that influence the organizational commitment performed by employees in healthcare institutions, and its repercussion on human resources management, objectives and consequences for the organization.

### 1.1. Organizational Commitment in Healthcare Institutions

Organizational commitment encompasses a series of behaviors performed by employees that lead them to undertake efforts for the good of the institution, a yearning to remain in it, and accepting its goals and values [14]. This definition, coined by Porter and Lawer in 1965, was updated by Greenberg and Baron [15] in 2008, in which they describe organizational commitment as the level of identification that an employee feels with the organization in which he/she works, which determines his/her level of commitment and intention to leave it. Organizational commitment can be analyzed from three different perspectives [16]: continuance or permanence commitment [17], which is based on the rootedness that the employee feels towards his institution caused by the small investments he has developed over time; affective commitment [18], which is more linked to the psychological rewards received by the employee, such as the recognition or support shown by other colleagues, so that the worker feels identified with his organization, accepting and sharing the company’s values and objectives; and normative commitment [17], which is related to the values of the worker himself and his responsibility with respect to his ethics in the workplace. Subsequently, other authors have continued to use this classification to distinguish the three types of organizational commitment, affective, continuance and normative [19]. Top et al. (2013) [20] rely on the studies of Meyer and Allen [16] where they highlight the importance of affective and normative commitment in improving organizational trust, also pointing out that it is still necessary to deepen this issue.

In the healthcare field, multiple research are found regarding organizational commitment, where it is linked to other factors related to human resources management. Mousa and Puhakka (2019) [21] delve into the relationship between responsible leadership and organizational inclusion, where they conclude that an environment of respect, equality and equity in the workplace contribute positively to the development of affective, normative and continuity engagement of physicians. The commitment to continuity or permanence is positioned as one of the critical aspects in healthcare institutions due to the high turnover of nursing staff. Older and more experienced nurses are more willing to stay in their jobs [22,23], although it also depends on the labor market opportunities in each case. Affective commitment in health care also has a clear particularity for this sector as it is positively related to job satisfaction [24] and trust in the organization. Normative engagement is possibly the most addressed in the healthcare literature, where it is related to demographic aspects of the worker, such as gender, place of origin and age, and also to the type of institution and work context.

Like other organizations, healthcare institutions try to reinforce the commitment to continuity or permanence in their workforces, mainly because of the difficulty of finding professionals in some cases [24,25,26] and because of the repercussions of mistakes made in healthcare [19]. However, this does not prevent most of the manuscripts analyzed in this research from addressing the problems of job dissatisfaction, stress, intention to leave, and other negative aspects of human resource management, indicating that this is a global concern in this sector. We can cite the work of Gorgulu and Akilli (2017) [27], where they describe a positive and significant relationship between levels of normative engagement and job satisfaction shown by healthcare workers, indicating how efforts made to reduce job burnout and psychologically supportive dynamics will improve motivation to provide better services, which will directly impact personal and institutional productivity.

### 1.2. Rationale and Structure of the Research

Publications on human resource management in healthcare institutions is booming, between 2015 and 2020, 224 manuscripts related to this subject have been published in the Web of Science database, which is the same number as between 1992 and 2015 [28]. Managers of healthcare centers face a significant challenge in the management of human resources in their organizations. The organizational commitment of employees and the factors that influence these links are positioned as a key factor in achieving the desired efficiency. In the publications analyzed, there is a common denominator related to job dissatisfaction, which develops in the form of intention to leave, stress, and lack of commitment to the organization.

In the face of this visible human resources problem in healthcare institutions, the systematic analysis carried out in this manuscript has allowed us to generate a model describing the relationships between the factors involved in the development of organizational commitment, the objectives of the company and the repercussions that can result from low organizational commitment caused by job dissatisfaction.

In this paper, following a systematic review of the literature, a model has been developed in which the main factors related to organizational commitment in healthcare institutions are positioned, where job satisfaction is positioned as the main moderating factor of organizational commitment, positively or negatively affecting the achievement of organizational objectives. The following sections develop the bibliometric analysis for the selection of the sample, design and analysis of the model, and its interpretation and discussion of the results obtained.

The document presented has the following structure: Introduction, where the justification for this research is presented; Methods, where the bibliometric techniques used are described, and materials and software; Results, in this section the most important issues resulting from the bibliometric analysis related to organizational commitment in healthcare institutions are described, also the model of organizational commitment in healthcare institutions is presented and developed, where the key aspects provided by the researchers in each case are detailed through a systematic review; ending with the Discussion and Conclusions.

## 2. Materials and Methods

The purpose of this research is to design a model that explains how organizational commitment affects Human Resource Management in healthcare centers. To this end, we propose to do so through a systematic review of the literature, analyzing in detail the influence of aspects related to organizational commitment and its consequences in the organization, in terms of the achievement of objectives or abandonment of the same. In order to focus the research on the trends with the greatest projection within the production of literature related to organizational commitment, we used bibliometric techniques. Publication in journals was used as the basic unit. Bibliometric analysis allowed us to examine the bibliographic material from an objective perspective, making it possible to organize the information within a specification of fields [29].

### 2.1. Materials

For the design of the bibliographic sample to be reviewed, the Web of Science (WoS) database [30] was chosen, as it is considered a rigorous source where the most prestigious scientific journals are found, and it gathers more than 115 years of research accumulating more than 171 million records. There are other more specific databases, but we did not consider them appropriate for the nature of this article. The optimal search criteria have been determined in order to obtain the greatest number of documents related to organizational commitment in health management systems.

The search criteria used were “organizational commitment” or “organisational commitment” and “health institutions” or “health system” or “healthcare” or “health care” or “health”. The fields used for the search were: article title, author keywords, keywords plus, and abstract. The search was restricted to the following indexes: science citation index expanded (SCI-EXPANDED); social sciences citation index (SSCI); emerging sources citation index (ESCI); art and humanities citation index (A&HCI); conference proceedings citation index-social science and humanities (CPCI-SSH); book citation index (BKCI); and science citation index expanded (CCR-EXPANDED). The year of publication was not limited in order to have the largest number of publications associated with this theme. Nor was the number of citations received restricted in order to include recent research that has not yet achieved the corresponding scientific impact.

**Figure 1:**
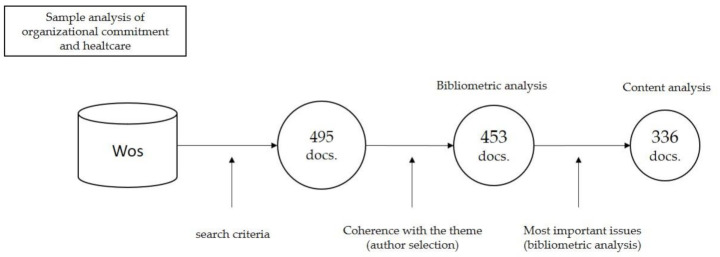
Sample analysis of organizational commitment and healthcare. Source: Prepared by the authors. Wos (Web of Science); Docs. (Documents).

The search was conducted in early January 2021 and yielded a total of 495 manuscripts from WoS, which, after a thorough review of the suitability and consistency of the papers with the research objective, 42 papers were rejected, resulting in a total of 453 (Figure 1). Subsequently, in order for the analysis to focus on the research trends leading the literature on organizational commitment in healthcare management systems, this set of documents was subjected to a bibliometric analysis and the documents that have had the greatest influence on the subject under study were extracted, resulting in a set of 336 documents, which will form the final sample. As will be seen in the following sections, this sample has proved to be remarkably suitable, both from a quantitative and qualitative point of view.

### 2.2. Software

The software used for the bibliometric analysis was SciMAT. This software application allows the construction of strategic maps and thematic networks with respect to a set of documents. The unit of analysis used was the author’s keywords and the keywords plus corresponding to the publication source. The choice of this software is due to the fact that, although there are many tools that allow scientific mapping, see Bibexcel, CiteSpace II, Co PalRed, IN-SPIRE, Gephi, VantagePoint, or VOSViewer, among others, SciMAT [31] synthesizes most of the advantages of the existing tools, and allowing longitudinal analysis, the essential objective of this work. The configuration was as follows: author keywords and source keywords were used as the unit of analysis. To create the networks, co-occurrences were used. To normalize the network, the equivalence index was used as the similarity measure. Finally, to create the scientific map of the topics and their networks, the single-center clustering algorithm was used.

## 3. Results

**Figure 2:**
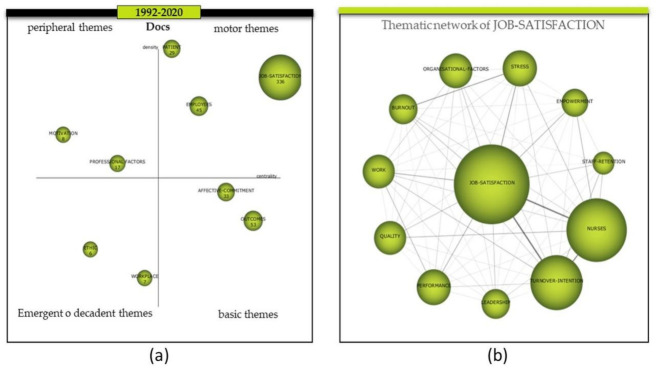
Evolution of the themes period 1992–2020 and thematic network of the cluster job-satisfaction. (**a**) Evolution of the themes period 1992–2020. (**b**) Thematic network of the cluster job-satisfaction. Source: Prepared by the authors on the basis of SciMAT data.

In order to find elements to create a model to explain organizational commitment in healthcare management systems, as detailed in the methodological section, a bibliometric analysis was carried out on the sample (453 documents). The time horizon used was that which covers all scientific production relating organizational commitment and healthcare institutions (1992–2020). The results provided by the strategic map of the bibliometric study (Figure 2a), place the job-satisfaction cluster with 336 documents as the main driving theme, i.e., as the theme with the highest centrality and density of all the literature in the sample. This means that the topics integrated in this cluster (Figure 2b) are the ones that have led the subject under study.

With regard to the members of the job-satisfaction cluster, burnout, empowerment, leadership, nurses, organizational-factors, performance, quality, staff-retention, stress, and turnover-intention are found. In terms of the most researched themes together, the results suggest that job-satisfaction was related to turnover-intention, nurses, stress, work and performance; nurses to turnover-intention and quality; and burnout to stress (Table 1). Based on the leadership of the job-satisfaction cluster, the 336 documents in its thematic network are taken as a sample for the systematic literature review. It is necessary to emphasize that, although the cluster takes the name of the most central theme, in this case job-satisfaction, it should not be forgotten that organizational commitment is the theme that unites the literature sample, therefore, this will be the central axis from which the different underlying themes emerge as results of our systematic analysis.

### 3.1. Proposed Model of Organizational Commitment in Health Organizations

**Figure 3:**
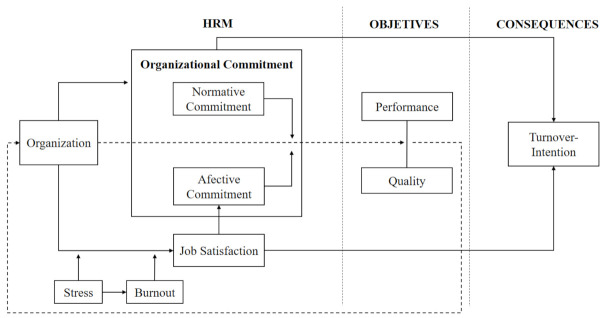
Organizational commitment model in healthcare organizations. Source: prepared by the authors.

From the analysis of the documents, results were obtained that were oriented to explain, from a transformational leadership approach, the importance of organizational commitment as a key aspect in the management of human resources of health personnel, and how this is fundamental to achieve the levels of performance and quality established by the health centers. On the other hand, the literature suggests that organizational commitment is moderated by job satisfaction. Moreover, job satisfaction is in turn affected by factors such as stress and burnout syndrome, which have a negative influence and can have decisive consequences on the work relationship, even leading to a situation of intent to leave the job (Figure 3).

### 3.2. Interpretation of the Proposed Model

#### 3.2.1. Organizational Commitment

Organizational commitment has been shown to be a key aspect of the work of health professionals, particularly nurses. Cho et al. [32] found that it was critical in providing better patient care and in ensuring organizational outcomes. Organizational commitment is also closely linked to work engagement and vocation in nursing work [33].

Organizational commitment has been shown to be a mediating factor that contributes to improved job satisfaction despite high job demands, especially in people over 45 years of age [34]. This variable makes the resources invested more efficient in improving job satisfaction and reducing the intention to quit [35]. It has also been shown to be effective in reducing employees’ long-term resistance to change [36].

All these factors become key to improving job performance. Negussie and Berehe [37] found that organizational commitment, job satisfaction, and work experience have been shown to be significant predictors of job performance in nursing professionals.

##### Factors that Enhance Organizational Commitment

In the development of organizational commitment there are numerous variables that have been found to be related, including demographic variables: age [38,39,40], educational level [40,41,42], gender [39], and nationality [38]. On the other hand, personal variables that enhance organizational commitment include: self-perception of effectiveness [43] and work ethos [44,45].

With regard to job-related variables that have been shown to contribute to improved organizational commitment, job satisfaction appears to be central to understanding this construct [41,46,47,48,49,50]. Additionally, other relevant variables are: adequate working hours and breaks [32], improved professional ethics and decreased ethical conflicts [51,52,53], work-related quality of life [54], years of service [39], salary satisfaction [46,55], task elaboration with identity, meaningfulness, autonomy and interpersonal [56], patient-centered care [46,57], job security [46]; organizational incentives [58], working in a private hospital [59], growth opportunities [60], and professional and continuing education opportunities [46,57,61].

At the organizational level, there are several aspects that management and human resources departments could modify, such as: development of an ethical and positive organizational climate [43,62,63], participative climate [64], organizational support and friendly relationships in the workplace [46,65], staff empowerment [66,67,68,69,70,71], psychological capital [72], cultural orientation [10,46,73], and organizational culture [41].

It is equally important to pay attention to the characteristics of superiors. Fundamental is leadership behavior [61], which should be based on authenticity [74], and be spiritual [75], transactional [66,76], health-oriented [77], and task-oriented [78]. Communication with superiors [79,80], quality of supervision [55,81], mentoring relationship [82], and praise and recognition by superiors [60,61,83] are also important.

Finally, attention must also be paid to the relationship between the organization and the individual, which is fundamental to understanding organizational commitment. In this regard, trust in the organization [20,47,49,50], professional commitment [58], and favorable perception of internal marketing [84] have been shown to be significant.

An easily modifiable aspect that contributes to the improvement of nurses’ organizational commitment and job satisfaction is their participation in the review and improvement of their performance appraisal process [85,86].

Physicians tend to have lower levels of organizational commitment than other health care workers, regardless of country, personal characteristics, type of work, or place of employment [87]. However, they tend to be more satisfied with their work than nurses in the same hospitals [88]. In their case, the variables related to organizational commitment are: age and job satisfaction [87]. On the other hand, nurses tend to be more satisfied with their work climate and more committed to their organization [43].

##### Factors Undermining the Development of Organizational Commitment

With regard to factors that negatively affect the development of organizational commitment we can highlight: presenteeism [89], stress [65,81], workload [90], bullying and harassment in the workplace [91], being a general practitioner [81], poor consultation with colleagues [81], conflict with organizational goals [58], burnout [70], supervisor incivility [70], organizational and superior cynicism [70,92], and abusive supervision [93]. It is also important to note how professional competence does not seem to have shown any impact on the development of organizational commitment [94].

##### Affective Commitment

Affective commitment is one of the three types of organizational commitment reported in the literature, and implies that the employee remains in the organization because he/she wants to, developing when the employee feels appreciated by the company and considers that the company meets his/her expectations and basic needs [95]. This type of commitment is promoted by perceived support from the organization, both from supervisors and coworkers [96,97], and by a climate of organizational justice [98], a trust in the organization [99] an adequate flow of information within the organization [100] and access to empowering factors, feeling that they can influence the organization in which they work [101]. This affective engagement has an impact on greater organizational inclusion [21], the development of networked behavior within the organization [102], better quality of care provided, and higher organizational performance [98,103]. Affective commitment is, in fact, that by which the relational psychological contracts developed by nurse managers are governed [104].

##### Normative Commitment

Normative commitment, which is largely based on the ethics demonstrated by the worker and compliance with organizational standards and policies [16,20], is present with greater significance in healthcare institutions relative to other organizations. Increased job satisfaction and normative commitment of nursing staff is directly linked to reduced intentions to leave [105,106]. Gambino [22] indicates that the strongest indicator regarding the intention to stay in the job of nurses in healthcare institutions is normative commitment, which is also reinforced as a function of the older age of the workers, and therefore recommends promoting normative commitment in younger nurses. Along the same lines, Gellatly [107] analyzes the relationship between intention to quit and high levels of normative and affective commitment on the part of nurses.

The rest of healthcare workers have also been analyzed in terms of organizational commitment and job satisfaction [27,108], where the influence of other factors such as job burnout, psychosocial support and motivation are described, and their relationship with normative commitment.

We understand that normative commitment is shown in a particular way in healthcare institutions, and that together with affective commitment make up the two basic sources of organizational commitment in these organizations, several authors recommend potentiating these factors in the search for greater personal and institutional productivity, and for the retention and attraction of new staff [27,106].

#### 3.2.2. Job Satisfaction

The available literature on job satisfaction among health professionals, and in particular nurses, is extensive and, in the last 5–10 years, has become even more extensive. Improved job satisfaction is relevant insofar as it is mediated by improved productivity and better service delivery [109,110].

Job satisfaction depends on numerous variables, starting with demographic variables such as age [111,112,113], marital status [112], nationality [111,112], educational level [42,113], being male [88,90], ethnicity [90], or psychological capital [72].

Regarding particular job conditions, some of the most relevant variables in the development of job satisfaction are salary [111,112,114], years of experience [112,113], being administrative staff [115], work shift [111], professional and continuing education opportunities [61], clinical autonomy and personal development [114,116], physical work environment [12], and spirit at work [45].

Some of the job factors that decrease job satisfaction are role conflict and role ambiguity [113,117,118], job stress [113,117,119], underestimation of job demand stressors [120], and burnout [70].

Additionally relevant are the characteristics of the organizational climate, with variables that promote job satisfaction being the development of an ethical work climate [52,63,121], organizational justice [122], development of a participative climate [64,116,123], company cultural values [73], use of information technology [124], workplace bullying and harassment [91], and structural empowerment [71,125,126].

In relation to the organization, the relationship developed with the organization is important, being the key variable to understand job satisfaction, organizational commitment [50,113,117,118,119,127,128,129], and professional commitment [113].

The relationship that develops with colleagues and superiors is also important. Regarding superiors, they should promote leadership behavior [61,129], in particular it seems advisable to develop spiritual leadership [75] and/or task-oriented leadership [78]. Likewise, recognition and praise from superiors [60,61,83,123], ethical behavior by leaders [109], and mentoring relationship [82] are also recommended. Factors that decrease satisfaction include supervisor incivility [70], and organizational cynicism on the part of superiors [70,92].

Regarding the relationship with colleagues, the quality of relationships at work [114,123], team cohesion [116], and team empowerment [130] are valued.

##### Factors Negatively Influencing Job Satisfaction. Effects of Stress

In relation to stress and job dissatisfaction we observed that the literature mostly supports a direct and positive relationship between the two ideas. Health care is a fertile field for service research and, due to the fact that staff suffer from physical and emotional stress, how to reduce burnout among health care staff is an emerging and important research question. Job stress and burnout have been positively associated with intention to quit, which may create more stress on other staff due to increased workload [131]. 

Velando-Soriano et al. [132] consider stress as the result of suffering burnout syndrome in the healthcare context, composed of three dimensions: emotional exhaustion, depersonalization, and decreased personal achievement. Jurado et al. [133] showed that burnout syndrome is significantly and negatively related to all factors of emotional intelligence, self-efficacy, and perceived social support. The risk of burnout is higher in younger people and in professionals with permanent employment.

Ethical climate and collegial solidarity also affect stress and organizational commitment [52,134] in healthcare with associations found between ethical climate and job satisfaction, moral distress, and intentions to leave. Emotional intelligence could be a mediating factor in the impact of job growth satisfaction and job stress but contrary to expectations, the emotional Intelligence factor did not significantly predict job stress [135].

Inconclusive results such as that of Somers et al. [5] who investigated employee well-being profiles using six well-being profile groups based on relative stress levels or an unexpected result, that of work engagement in risky and stressful professions [136], which gave the comparative result that nurses were the least engaged.

Innovation at work is another important factor in eliminating job stress. In this sense, stressors arising from role conflict and role ambiguity have a negative effect [137] while the implementation of innovative practices related to clear job design has the opposite effect [138]. Additionally, physical factors such as light can be a source of stress in healthcare employees and the role of lighting in mitigating patient stress has been studied by McCunn and Wright [139]; contrary to the intention of the facility, participants did not perceive circadian lighting to have greatly improved their stress levels, concentration, mood or fatigue at work.

Chen and Chen [8] explore the possible antecedents and consequences of nurse burnout and examine the moderating effects of personal traits and the issue of work environment. The results reveal positive causality between job stressors and nurses’ burnout, while supervisor support is negatively related to burnout.

Yang and Fry [75] examined the extent to which spiritual leadership reduces burnout among medical laboratory staff while positively influencing organizational commitment, work unit productivity and employee life satisfaction. The results revealed that spiritual leadership had direct and mediating effects on psychological distress, poor health, and negative work attitudes. Based on the transactional stress model of the moral self, Wang et al. [53] analyzed psychological strain as a key mediating mechanism that channels the negative relationship between ethical conflict and organizational commitment.

##### Factors Negatively Influencing Job Satisfaction. Effects of Burnout Syndrome

Emotional exhaustion or burnout is common in nursing professionals [140], and tends to become more frequent in the face of high work demands [141]. Several factors prevent burnout, such as spiritual leadership [75], transformational leadership [142], affective engagement [9], empowerment [71], demographics, work environment, or work attitudes [140,143]. The risk of burnout is higher among younger people and professionals in permanent employment [133]. With respect to job position emotional exhaustion is higher in nurses, administrative staff, and technicians [144]. Social support from supervisors and coworkers, stress management, and self-efficacy have been shown to be key factors in its prevention [132,133].

In this regard, when communicating during nursing supervision, Kim and Lee [145] found that relationship-based communication protects against burnout and turnover, while work-focused communication promotes burnout. Consequences of burnout include physical health problems, poor job performance, low organizational commitment, and intention to leave [143].

In order to combat the effects of burnout, Permarupan et al. [146] stress the importance of psychological empowerment of nurses, which is related to an improvement in the quality of working life (adequate remuneration, safe and healthy working conditions, social integration, etc.). With regard to the development of empowerment in nurses, Gholami et al. [147] highlight the importance of improving the organizational climate.

#### 3.2.3. Turnover Intention

##### Factors Favoring the Intention to Quit in the Health Sector

Intention to leave has been related to a number of variables, although one of the most frequently studied is that which relates it to organizational commitment [10,106,107,108,109,110,111,112,113,114,115,116,117,118,119,120,121,122,123,124,125,126,127,128,129,130,131,132,133,134,135,136,137,138,139,140,141,142,143,144,145,146,147,148], being in some studies the only factor implicated [149]. Along these lines, several authors have also mentioned affective and normative commitment [22,105,106,107,108,109,110,111,112,113,114,115,116,117,118,119,120,121,122,123,124,125,126,127,128,129,130,131,132,133,134,135,136,137,138,139,140,141,142,143,144,145,146,147,148,149,150,151,152,153,154,155], as a key aspect in understanding this phenomenon.

Factors predicting intention to quit in healthcare include demographic variables, such as being younger [22,90,153,156], being moderately or well educated [42,157], and being female [157]. Personal variables that encourage quitting include poor subjective health [158], availability of other job opportunities [150], and controlled motivation [159].

Regarding job-related characteristics, we should highlight job dissatisfaction [113,148,152,156,157,158,160,161,162], which has been widely collected in the literature. Other variables in this related category are: working in an intensive care unit [153], job stress and job burnout [70,150,154,162], also discrimination and unequal treatment based on nationality [163], experiences of threat or violence by patients [164], poor family reconciliation [150,152,154,165], and those factors related to working time (part-time work, overtime, or long commuting time) [148,157].

We must also highlight the actions of superiors and the organization, which can also encourage quitting. Organizational cynicism on the part of superiors [70,92], abusive supervision [93], and supervisor incivility [70] have all been shown to encourage quitting among health professionals.

##### Factors Favoring the Intention to Remain in the Workplace

Factors that have been shown to increase the likelihood of turnover in the health sector include the development of opportunities for continuing medical education, for which high expectations also need to be raised [40,114].

Demographic and personal variables that have been shown to promote health professionals to stay in their jobs include: marital status [106], empowerment [70,151], autonomous motivation [159], and cultural orientation [10].

Job-related variables include: salary and promotion opportunities [90,156,157], job quality [105], achievement and job security [155,160], length of service [60], the use of information technology [124], and job embeddedness [165].

Finally, with regard to the characteristics of the organization and the relationship between the two, in order to avoid quitting, the following should be promoted: an ethical climate [52], organizational justice [122], good physical working environment [12], organizational culture and values [148], person-organization fit [166], and coworker relationships and sense of community [156,167]. It is also important to promote servant leadership behavior [168], supportive and communicative leadership [154], and ethical leadership [169].

## 4. Discussion

The proposed model, resulting from the bibliometric analysis of the literature, places organizational commitment in a central place. Similarly, job satisfaction is a human resource management practice necessary for the achievement of performance and quality objectives. Stress and burnout appear as moderating factors of the model, the main consequence being to avoid abandonment in the health management system.

From studies on improving organizational commitment and job satisfaction, and on preventing job burnout, turnover, and emotional exhaustion, the literature compiles a number of guidelines that should be taken into account by human resource management professionals in health-related areas.

First, it is important to highlight the traits of health managers related to better performance in their profession: higher degree of teamwork, communication within the team, and organizational commitment [170]. It is also important to pay attention to the practices of managers. Achievement-driven, rather than power-driven, motivation of managers in a healthcare context is positively correlated with the use of leadership behaviors, nurses’ job satisfaction, productivity, organizational commitment, and patient satisfaction [46].

In order to improve organizational commitment, occupational engagement, and growth satisfaction and well-being at work, Kanste et al. [171] recommend promoting the development of networks between primary and special health care units. In this sense it is important to promote innovative behavior generating positive perceptions of well-being [172] and staff empowerment [71].

To boost the performance and productivity of health care workplaces, the literature systematizes recommendations of high impact as taking into account the emotional intelligence of nurses when assigning shifts [173]. In fact, nurse manager capability has been associated with high emotional intelligence, which is also high amongst those in this role [174]. An organizational culture must be provided that enables nursing professionals to report medical errors [175]. Improving the quality of organizational culture, which affects the quality of work life, organizational commitment, job satisfaction, and employee turnover [176].

An open communication flow in the organization, the meaningfulness of the work and appreciation by superiors is connected to the quality of the service offered (Lampinen et al., 2017). In order to develop a high-performance work atmosphere, Boselie [177] recommends the development of employees (through general and skills training and task enrichment) and the promotion of employee participation (autonomy at work, participation in decision-making, etc.).

Among health professionals, turnover intention seems to depend on occupation. Thus, in the study by Hwang and Chang [178] turnover rates were highest among doctors, followed by paramedics, nurses, and finally administrators. There are numerous factors that encourage turnover in health workers, such as demographic factors (nationality, education, gender, and region), experience, availability of alternative employment [179], perception of human capital and level of staff training [26], and job integration [25].

Turnover intention is also related to the conditions and situations developed within the workplace. Laeeque et al. [180] found the importance of patient-inflicted violence in predicting staff turnover, although this relationship is mediated by stress and burnout. Other job-related factors include low pay [179], low job satisfaction [63,162,181,182], job stress [13,162], job burnout and poor work–life balance [162], racial and/or ethnic discrimination in the workplace [183], ethical conflicts [63,184], and poor citizenship behavior [185]. Other authors have also analyzed organizational variables and their impact on turnover intention, where we highlight the weight of low organizational justice [122], low organizational commitment [13,63,162,182,185,186], and low structural empowerment [69,71].

It is important to understand, however, that these factors are mediated by several variables, such as whether the staff is internal or external. In the study by Chiu et al. [187] it was found that commitment to the organization and management support seem to influence internal staff more, whereas perceived job stress affects external staff more. In the case of nurses who want to leave the profession, Flinkman et al. [11] suggest further investigation by including new variables as it is a hot and important topic.

Anomalously, in our analysis we did not find papers on continuity commitment as a central or important theme. Similarly, transformational leadership does not occupy a place of impact among the topics analyzed in the literature reviewed. This is the reason why it does not appear in the proposed model. Although we consider that it should be included as a strategy within organizational commitment.

As a result of the research objective set out in our work, we obtained a valid model to prevent turnover intention in healthcare workers worldwide. In this model, positive inputs to the organization (organizational commitment and job satisfaction) and negative inputs or moderating factors (stress and burnout) generate results in terms of turnover intention, which is what we try to use as feedback to generate new positive inputs to avoid bad results in terms of performance or quality. We believe that our model meets the expectations that healthcare organizations need in order to have a benchmark or guide for decision-making to avoid the abandonment of hospital staff.

Studies such as Akkaya [188] or Bosak et al. [64] support our model by including productivity and positive employee attitudes as variables for improving organizational performance. Their results corroborate our model by showing that the climatic level of employee engagement is positively associated with individual employee attitudes (i.e., job satisfaction and organizational commitment) and organizational effectiveness (i.e., shorter outpatient waiting times and higher quality of performance) as in the case presented by Mulki et al. [6]. The results provide practical insight into how supervisors play an essential role in alleviating staff burnout. We agree with Chen and Chen [8] in recommending supportive attitude and leadership effectiveness as effective management strategies. 

The objectives pursued in our model concerning performance and quality coincide with those studied by Cho et al. [32] recently. The performance of healthcare staff as an objective of our model has been corroborated by Nangoli et al. [168] by relating it to leadership practices or by Parveen et al. [189] with human resources practices of high commitment to the organization. 

Our generic model is also supported by Attia et al. [43] who compared job performance in nurses and doctors, with the former having better results in terms of job outcomes and job satisfaction. Additionally, in the study by Gostautaite [190] it includes age as a moderator of work outcomes. Quality of patient care is also a variable to be taken into account in our model and has recently been used by Khera et al., [191] or Plourde [192]. Issues that find a place in the perception of quality in our model are ethnicity, race, and equality in the treatment received in health care [193] and the quality of the work of nurse care managers [194].

We agree with Baxter et al. [195] that providing patient care that is cost-effective, accessible and of high quality is a challenge for governments and health care delivery systems around the world. Their analysis concludes that regardless of the type of hospital financing reform implemented, health care leaders described a complex process that required the following: organizational commitment; adequate infrastructure; human, financial and information technology resources; champions for change; and a personal commitment to quality care. Employee engagement and control theories propose that health workforce initiatives are critical to patient safety [196] highlighting the need to develop quality health research [197].

## 5. Conclusions

By creating and maintaining a positive and strong climate of engagement, hospital managers can address the productivity challenge that hospitals and healthcare institutions in general are currently facing, while improving the attitudes of their employees, which are central to the transformation process and ultimately underpin organizational success. The results highlight the role of atmosphere strength and underline its importance in future research and practice.

This study suggests that general self-efficacy and stress management act as protective factors against the likelihood of burnout. Thus, organizations should encourage transformational leadership training and coaching programs to promote workers’ organizational well-being and engagement [133].

Our results support the differential effects of job demands and stressors on the implementation of innovation in healthcare work [137]. Stressors stemming from role conflict and role ambiguity have a negative effect on job satisfaction, while the implementation of innovative practices related to clear job design has the opposite effect. Therefore, it is a matter of implementing HR practices that advocate a clear and coherent design that eliminates reasons for ambiguity and stress in carrying out the tasks of the healthcare worker’s job.

We agree with Valentine et al. [198] in incorporating group creativity and ethical values as a guarantee of greater job satisfaction. We also support the idea of Watt et al. [199] in involving staff in the process of cultural change, however, we disagree with Taghavi et al. [200] in the need to establish a human resource model for hospital administrators based on relationships with what they call “customers” (not “patients”). Finally, it is vital to believe strongly in the idea of transformational leadership, organizational commitment, organizational trust, and job satisfaction in the health care industry [47].

It would be convenient to include one more variable in the proposed model in the form of “staff retention”, i.e., in our model outcomes we included the most repeated term in the literature, which is “turnover intention” but we consider that the model could be enriched with the introduction of an additional outcome, which is “staff retention”. This will not only prevent staff from leaving the organization but also include all the necessary positive attitudes and human resource policies to be applied by management in order to keep staff in the organization. In this sense, it would also be convenient to include the variable “empowerment” as a contributor in the search for staff retention.

Similarly, it should be noted that the 336 documents finally selected for this systematic analysis only come from the WoS database. We are aware that there may be scientific literature of great interest not included in our sample; however, the results obtained indicate that the number of documents analyzed was sufficiently large. On the other hand, if studies published in other databases or non-academic books and journals have been taken into account for the introduction and approach of the research.

We also consider it a future line of research to include the role of supervisors in the model proposed. Taking into account the reality of this figure in healthcare organizations, it would be necessary to analyze their job profile and the extent to which they contribute to the intention to leave the organization. Although they have a difficult mediating role between the organization and the employees, their tasks should be analyzed and it should be seen how their functions can be channeled as facilitators in the retention of staff rather than as inciters to leaving, always complying with the performance and quality demanded by the organization.

## Figures and Tables

**Table 1:** Thematic network job-satisfaction 1992–2010. Relationships with a weight equal to or greater than 0.10.

No.	Node A	Node B	Weight
1	Job-Satisfaction	Turnover-Intention	0.36
2	Job-Satisfaction	Nurses	0.35
3	Nurses	Turnover-Intention	0.25
4	Job-Satisfaction	Stress	0.15
5	Burnout	Stress	0.15
6	Job-Satisfaction	Work	0.12
7	Job-Satisfaction	Performance	0.11
8	Nurses	Quality	0.10

Source: Prepared by the authors on the basis of SciMAT data.

## Data Availability

Not applicable.
